# Impact of precipitation on the prevalence of schistosomiasis mekongi in Lao PDR: Structural equation modelling using Earth observation satellite data

**DOI:** 10.1016/j.onehlt.2023.100563

**Published:** 2023-05-11

**Authors:** Emilie Louise Akiko Matsumoto-Takahashi, Takashi Kumagai, Kei Oyoshi, Yoshinobu Sasaki, Yousei Mizukami, Bouasy Hongvanthong, Paul T. Brey, Shigeyuki Kano, Moritoshi Iwagami

**Affiliations:** a Department of Tropical Medicine and Malaria, Research Institute, National Center for Global Health and Medicine (NCGM), Tokyo, Japan; b Graduate School of Public Health, St. Luke's International University, Tokyo, Japan; c Department of Parasitology & Tropical Medicine, Graduate School of Medical and Dental Sciences, Tokyo Medical and Dental University, Tokyo, Japan; d Earth Observation Research Center (EORC), Space Technology Directorate I, Japan Aerospace Exploration Agency (JAXA), Tsukuba, Japan; e Center of Malariology, Parasitology and Entomology (CMPE), Ministry of Health, Vientiane, Lao Democratic People’s Republic; f Institut Pasteur du Laos (IPL), Ministry of Health, Vientiane, Lao Democratic People’s Republic; g Parasitology Laboratory, Institut Pasteur du Laos (IPL), Ministry of Health, Vientiane, Lao Democratic People’s Republic

**Keywords:** *Schistosoma mekongi*, Climate change, Earth observation satellite, Lao PDR

## Abstract

Increasing attention is being given to the effect of climate change on schistosomiasis, but the impact is currently unknown. As the intermediate snail host (*Neotricula aperta*) of *Schistosoma mekongi* inhabits the Mekong River, it is thought that environmental factors affecting the area of water will have an impact on the occurrence of schistosomiasis mekongi. The aim of the present study was to assess the impact of precipitation on the prevalence of human schistosomiasis mekongi using epidemiological data and Earth observation satellite data in Khong district, Champasak province, Lao PDR. Structural equation modelling (SEM) using epidemiological data and Earth observation satellite data was conducted to determine the factors associated with the number of schistosomiasis mekongi patients. As a result, SEM identified 3 significant factors independently associated with schistosomiasis mekongi: (1) a negative association with mass drug administration (MDA); (2) negative association with total precipitation per year; and (3) positive association with precipitation during the dry season. Precisely, regardless of MDA, the increase in total yearly precipitation was suggested to decrease the number of schistosomiasis patients, whereas an increase in precipitation in the dry season increased the number of schistosomiasis patients. This is probably because when total precipitation increases, the water level of the Mekong River rises, thus decreasing the density of infected larvae, cercaria, in the water, and the frequency of humans entering the river would also decrease. In contrast, when precipitation in the dry season is higher, the water level of the Mekong River also rises, which expands the snail habitant, and thus water contact between humans and the snails would also increase. The present study results suggest that increasing precipitation would impact the prevalence of schistosomiasis both positively and negatively, and precipitation should also be considered in the policy to eliminate schistosomiasis mekongi in Lao PDR.

## Highlights

• The risk of schistosomiasis infection due to changes in precipitation was analysed.
• Earth observation satellite data and epidemiological data were used in the analysis.
• An increase in the total yearly precipitation decreased the number of patients.
• An increase in precipitation in the dry season increased the number of patients.
• Intensive measures against schistosomiasis are needed especially in the dry season.

## 1 Introduction

Schistosomiasis, a life-threatening infectious disease caused by blood-borne flukes of the genus *Schistosoma,* is one of the world's greatest neglected tropical diseases affecting humans and animals living in tropical and subtropical areas [1]. The parasite affects >240 million individuals globally, and the estimated global burden of schistosomiasis accounts for 3.4 million disability-adjusted life years lost annually [2,3].

Infection occurs when free-swimming larval forms of the parasite (cercaria), are released from the intermediate freshwater snail host into the water, then penetrate the skin while in contact with contaminated water during agricultural, fishing, other occupational, and recreational activities [4]. The disease causes non-specific, but disabling systemic morbidities including anaemia, malnutrition, and impaired childhood development, which can lead to a reduced ability to learn and work [5]. Although these symptoms are usually recovered by pharmaceutical therapy, a severe chronic phase of the disease may result in death. At present, the World Health Organization (WHO) is focused on reducing the number of schistosomiasis patients through mass drug administration (MDA), a periodic (once every 1 or 2 years), large-scale population treatment with the isoquinolinone drug praziquantel to suppress morbidity [2].

*Schistosoma mekongi* is one of the most geographically restricted species, which focally, but can be found in relatively large numbers in limited areas near the border between Lao People's Democratic Republic (PDR) and Cambodia [6]. Since the discovery of this disease in 1957 in the Khong district of Champasak province, southern Lao PDR, schistosomiasis mekongi continues to be a health concern in the lower Mekong basin [7]. This disease is caused by the parasite *S. mekongi*, with the freshwater snail *Neotricula aperta* serving as an intermediate host [8]. *N. aperta* is particularly prevalent in areas of rocky banks along the Mekong River Basin [9].

Despite the annual MDA of praziquantel and health education over the last two decades, the question remains as to the most effective approach to future control to eliminate schistosomiasis mekongi [10]. Although the discovery of schistosomiasis mekongi in Lao PDR occurred in 1957, because of political upheaval, the endemic situation become worse, and no control program was implemented until the 1980s [6]. MDA was started in 1983 by the Ministry of Health, Lao PDR, with funding and technical assistance from WHO. The MDA focused on 60,000 inhabitants living on Khong Island and outlying villages in Khong and Mounlapamok districts, Champasak province. Although the prevalence exceeded 50% (by Kato-Katz methods) at the baseline assessment, after MDA, the prevalence rapidly decreased year by year. MDA was stopped once in 1998, but resurgence of this disease was confirmed within a short period of time, and yearly MDA resumed in 2006 and has continued until 2023.

Furthermore, over the past 20 years, increasing attention has been given to the effect of climate change on schistosomiasis. The impact is currently unknown partly because of the diversity of the species of both the snails and the *Schistosoma* spp., but both warrant examination [11]. Little research has been reported on the impact of climate change on schistosomiasis, most of which primarily used temperature as the sole driving factor [12]. Most studies were conducted in China and pertained to *S. japonicum* [13,14], with some conducted in western Africa, mainly for *S. mansoni* [15,16].

To our knowledge, no research has been conducted to analyse the impact of climate change on *S. mekongi*, even though at least 60,000 inhabitants are being threatened in the endemic areas [8]. In addition to temperature, as the snail host lives in the Mekong River, precipitation affecting the water level of the river should be also an important factor. Therefore, the aim of the present study was to assess the impact of climate change (precipitation and land surface temperature (LST)) on human schistosomiasis mekongi using Earth observation satellite data in Khong and Mounlapamok districts, Champasak province, Lao PDR.

## 2 Materials and methods

### 2.1 Study area

**Fig. 1:**
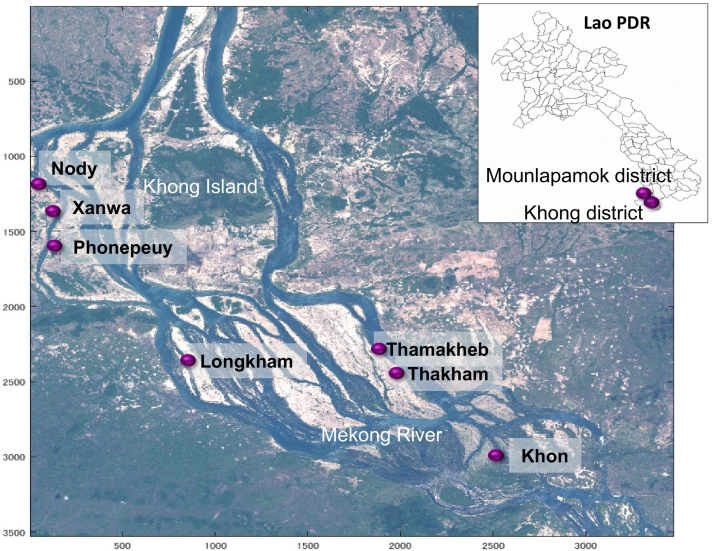
Study area. The risk of schistosomiasis mekongi infection in Lao PDR is present in Khong Island, its southern islands and outlying villages in the Mekong River, Khong, and Mounlapamok districts. The map also shows the seven sentinel villages selected by the Lao Ministry of Health, Lao PDR and WHO. The satellite image was taken by the AVNIR-2 onboard ALOS on 2 April 2009.

The present study was conducted in Khong and Mounlapamok districts in Champasak province, the epidemic area of schistosomiasis mekongi in Lao PDR (Fig. 1). The area includes Khong Island (14.142084 N latitude, 105.822112 E longitude based on the geodetic datum WGS84) and the seven sentinel villages selected by the Lao Ministry of Health, Lao PDR and WHO (Khong district: Phonepeuy, Longkham, Thamakheb, Thakham, and Khon; Mounlapamok district: Nody, Xanwa). Khong Island is the largest of the Mekong islands and is some 25 km north of the border with Cambodia. Approximately 60,000 people (mostly farmers and fishermen) live in and around the island [8].

Khong Island and its southern islands are endemic with schistosomiasis mekongi. During the low-water period (February to May), many islets around Khong Island are observed and capture sections of the river as shallow streams. These shallow regions form ideal habitats for *N. aperta* and their food, such as diatoms and filamentous green algae [8].

### 2.2 Data sampling

To assess the impact of precipitation on human schistosomiasis mekongi in Khong and Mounlapamok districts, epidemiological data on schistosomiasis mekongi was collected from the Ministry of Health, Lao PDR. The present study used the compiled data set [17] of all of the available historical data on the prevalence of schistosomiasis in Lao PDR during 2002 through 2012, which were collected in (1) the National Center of Malariology, Parasitology and Entomology, (2) National Institute of Public Heath, and (3) Institut Pasteur du Laos. This data was collected using the Kato-Katz microscopy method, which is not as accurate as it could be for low-intensity infections. Infection intensity is based on the number of *S. mekongi* eggs per gram (EPG) of fecal specimens: light 1–99, moderate 100–399, and heavy 400<. To compensate for this shortcoming, two consecutive fecal samples per person were collected. Fecal sample surveys were conducted annually before the MDAs were conducted. The survey targets 200 people from each village (random sampling). Besides, residents of this endemic areas (aged 5 to 65) are treated with praziquantel once a year because they are eligible for the annual MDA. The same age group was also the subject to the fecal examination while their adherence to the medication was not monitored.

Precipitation and LST data during 2002 through 2016 observed by Earth observation satellites was acquired from the Japan Aerospace Exploration Agency (JAXA) Public-health Monitor and Analysis Platform (JPMAP) [18] developed by JAXA. JPMAP utilizes GSMaP [19] provided by JAXA as precipitation data and the MOD11 and MYD11 products [20] provided by the National Aeronautics and Space Administration (NASA)/United States Geological Survey (USGS) as LST data. The users are able to search and retrieve data for a selected period or area (point, rectangular administrative area) via a user-friendly website (https://www.jpmap-jaxa.jp/jpmap/en). All of the geographical data was analysed using Quantum Geographic Information System (QGIS) 2.18.22 (Development Team - Open Source Geospatial Foundation Project, 2018).

### 2.3 Statistical analysis

Structural equation modelling (SEM) analysis was used to identify the factors associated with the prevalence of schistosomiasis mekongi. The correlation of all variables was examined, and a path model was built based on the results of bivariate analysis. The fit of the model was examined in terms of degree of freedom (df), chi-square (CMIN), comparative fix index (CFI), and root mean square error of approximation (RMSEA) [21]. According to conventional criteria, a good fit was indicated by CMIN/df < 2, CFI > 0.97, and RMSEA <0.05, and an acceptable fit by CMIN/df < 3, CFI > 0.95, and RMSEA <0.08. A value of *p* < 0.05 was considered to indicate statistical significance. All statistical analyses were conducted using SPSS version 24.0 and Amos 24.0 (SPSS Inc., Chicago, IL, USA).

## 3 Results

### 3.1 Precipitation, land surface temperature, and earth observation satellite images

**Fig. 2:**
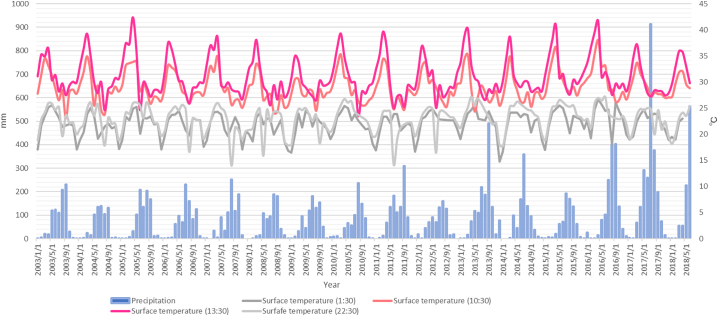
Precipitation and land surface temperature in Khong district (year 2003 to 2018).

**Fig. 3:**
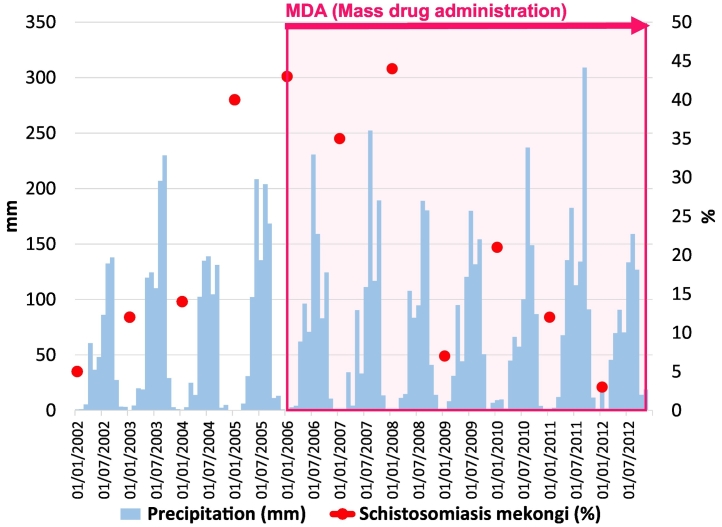
Precipitation, prevalence of schistosomiasis mekongi, and the duration of mass drug administration (MDA) in Khong district. The precipitation data was obtained from GSMaP (Global Satellite Mapping of Precipitation) (10-km resolution). Data on the prevalence of schistosomiasis mekongi and MDA are based on a WHO mission report [17].

From 2003 through 2016, the total annual precipitation in the endemic areas of schistosomiasis mekongi increased by 508 mm, and average annual LST at 13:30 rose by 1.1 °C (Fig. 2). The total precipitation and average LST continued to rise until 2016. In 2017 and 2018, with a doubling of annual total precipitation compared with year 2016, the average LST decreased. (Fig. 3).

**Fig. 4:**
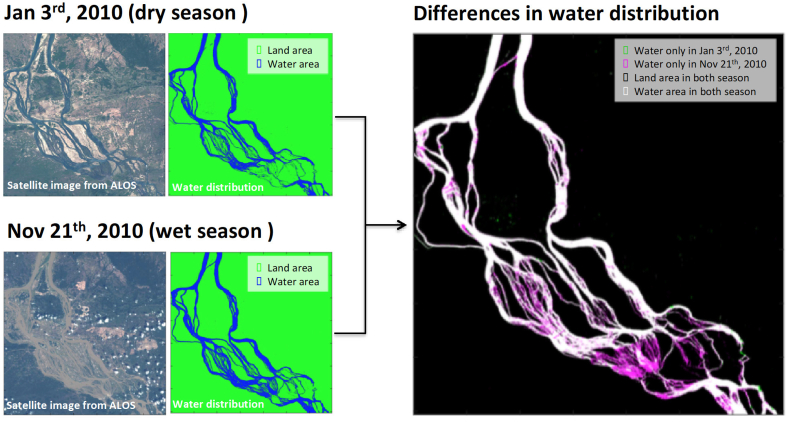
Differences in water distribution of the Mekong River during the wet season and dry season in Khong and Mounlapamok districts (year 2010). Water covered the area on 3 January (dry season) and 21 November 2010 (wet season), and the seasonal difference between them was delineated from satellite imagery observed by the AVNIR-2 onboard the ALOS with spatial resolution of 10 m. Most schistosomiasis infections in humans occur at the end of the dry season (between April and June) when matured snails are observed. Places of infection places are considered to be areas under water in the wet season that become dry land during the dry season (shown in pink). During the dry season, the temperature is the highest of the year. The water level of the Mekong River is low, the flow is slow, and therefore flooding activity of the people is the highest in the year. (For interpretation of the references to colour in this figure legend, the reader is referred to the web version of this article.)

In the analysis using the Earth observation satellite images, it was possible to overview the region of risk of the infection of *S. mekongi*, and the suitable snail habitat, by clearly indicating the difference in water area between the dry and wet seasons. Fig. 4 shows the water-covered area on 3 January (dry season), 21 November 2010 (wet season), and seasonal difference between them delineated from satellite imagery observed by the Advanced Visible and Near Infrared Radiometer type-2 (AVNIR-2) onboard the Advanced Land Observation Satellite (ALOS) satellite. Additional analysis found that the water-covered area of the dry season differed slightly by year. The Normalized Difference Vegetation Index (NDVI) [22] was calculated from red and near infrared imagery of AVNIR-2. The NDVI ranges from −1 to 1, and as bodies of water are normally indicated by a NDVI of <0, we regarded areas with a NDVI of <0 as bodies of water.

### 3.2 Impact of precipitation on schistosomiasis mekongi

**Fig. 5:**
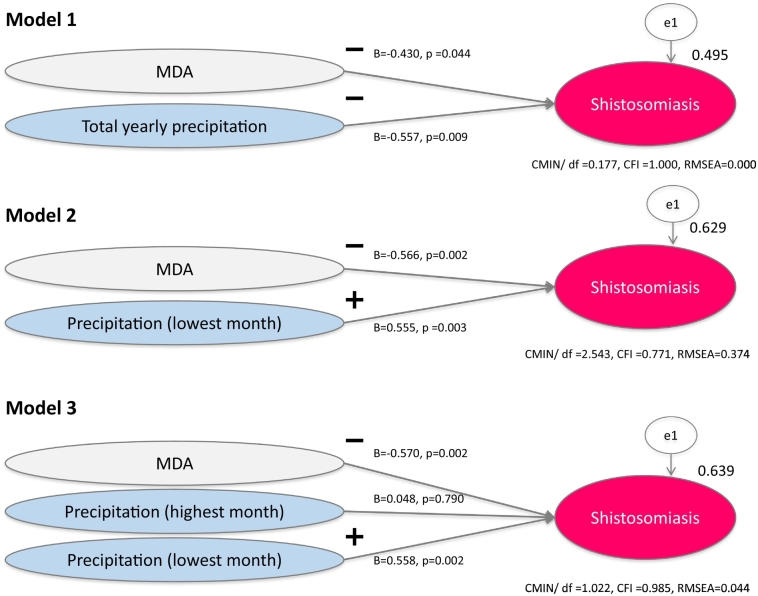
Impact of precipitation change on schistosomiasis mekongi during 2002 to 2012 in Khong and Mounlapamok districts, Lao PDR. CFI = comparative fix index; CMIN = chi-square; MDA = mass drug administration of praziquantel; RMSEA = root mean square error of approximation.

Using the epidemiological data and the Earth observation satellites data shown in Fig. 3, three hypothetical SEMs were built to examine the impact of precipitation on schistosomiasis from 2002 through 2012 (Fig. 5). The hypothetical SEM was built based on bivariate analyses and was selected from several models with consideration of the fitness between the data and the model, and the usability obtained from the results.

All of the models highly fitted the data. In model 1, the following directional paths were drawn: MDA to schistosomiasis and total precipitation per year to schistosomiasis. Namely, regardless of the presence of MDA, as the amount of precipitation increased, the number of schistosomiasis patients decreased (*p* < 0.05). In model 2, similar directional paths were drawn: MDA to schistosomiasis and precipitation in the month with the least precipitation per year to schistosomiasis. In other words, regardless of the presence of MDA, when precipitation in the month with the least precipitation per year increased, the number of schistosomiasis patients also increased (*p* < 0.05). Moreover, as shown in model 3, precipitation in the month with the most precipitation per year had no impact on the number of schistosomiasis patients.

## 4 Discussion

The present study was conducted to determine the impact of climate change on schistosomiasis mekongi. As a result, SEM determined three independent factors: (1) a negative association with MDA; (2) a negative association with total precipitation per year; and (3) a positive association with precipitation in the dry season (*p* < 0.05).

First, regardless of the presence of MDA, increasing yearly precipitation significantly reduced the number of schistosomiasis mekongi patients, likely because if the water level of the Mekong River is high, the density of cercaria of *S. mekongi* in the water decreases. Therefore, the number of schistosomiasis mekongi patients should also have decreased. Our results concur with another study assessing the impact of precipitation on *S. mansoni* transmission in Ethiopia, which also found a significant negative correlation between monthly precipitation volume (derived from rain gauge or satellite data) and the number of patients at lags of 1 and 2 months [23]. Although the exact mechanism is not clear, they suggested a possible explanation for this finding in that increasing precipitation might create an environment that is disadvantageous for the survival of snail and cercaria as well as transmission of cercaria from the snail to human.

Second, regardless of the presence of MDA, an increase in precipitation in the dry season significantly increased the number of schistosomiasis mekongi patients in the endemic areas. Most schistosomiasis infections in humans occur at the end of the dry season (from October to April) when mature snails are observed [6,8]. Infection in humans is known to occur mainly during the low-water period (dry season) in the Mekong River basin, and places with high risk of infection are considered to be areas under water in the wet season and but that become land during the dry season. The temperature during the dry season is the highest of the year. As the water level of the Mekong River is low, the flow is slow, and therefore the density of snails [24,25] and cercariae are considered highest throughout the year. The present study showed that a decrease in precipitation in the dry season significantly decreased the number of schistosomiasis mekongi patients. This is probably because when the water level is too low, the snail habitat narrows, the river temperature increases too much for survival of the cercaria of *S. mekongi*, and the risk of contact between human and the cercaria may also be decreased. Moreover, the present study found yearly differences of the area of water coverage during the dry season. This result indicates the possibility that the area of risk may change per year. As the satellite images clearly identify the areas of risk, these images could play an effective role in risk prediction.

Although it should be obvious, the statistical analysis of the present study proved that MDA had significantly reduced the number of schistosomiasis patients, although MDA does have some limitations. In 2021, at least 251.4 million people living in the tropical and subtropical area were estimated to require preventive treatment for schistosomiasis, of which 75.3 million people (30.0%) received preventive chemotherapy for schistosomiasis by MDA [26]. However, it is also known that the coverage rate reported by governments or international agencies is higher than the actual proportion of the population who actually receives the drug and swallows it [27,28].

In addition, pregnant and lactating women are generally less likely to receive the treatment [29,30]. In fact, the majority of adult women in the endemic areas in Khong and Mounlapamok districts are excluded from MDA because the guideline for praziquantel usage in Lao PDR does not allow prescribing it to pregnant and lactating women. However, WHO recommendations and a recent study showed that the administration of praziquantel to pregnant and lactating women is safe and needs to be clearly translated into national policies in the endemic countries [31].

There is a demand to promote effective health education to achieve a high compliance rate with MDA and prevent new infections with *S. mekongi* until the day schistosomiasis is eliminated. Indeed, the Community-led Initiative to Accelerate Elimination of Schistosomiasis with Water Supply, Sanitation and Hygiene (CL-SWASH) plan is now ongoing by NAM SAAT (The Center for Environmental Health and Water Supply) under the Ministry of Health, Lao PDR, in some selected villages in endemic areas of the country. This type of program should be expanded to other endemic villages in Khong and Mounlapamok districts.

Furthermore, Lao PDR should be cautious of the growing number of tourists visiting Khong district, where they enjoy several activities, such as visiting the Khone Phapheng Waterfall, the largest waterfall in Southeast Asia, and ecotourism, including watching Mekong dolphins by boat and kayaking in the Mekong River. While economic development of such travel industry is important, it might also be necessary to implement awareness activities that inform travelers about the transmission, prevention, and treatment (i.e. it is a treatable disease) of schistosomiasis mekongi.

Finally, the results of the present study suggested that the Earth observation satellite data (precipitation and satellite images) could provide effective evidence-based results for additional measures against schistosomiasis mekongi in Lao PDR. The precipitation data could predict an increase of the number of schistosomiasis mekongi patients, and the satellite images could provide detailed information on areas of infection risk. Using these data, it would be possible to conduct additional measures such as awareness-raising activities during the high-risk period in the areas of high risk.

Moreover, in an era of climate change, the Earth observation satellite data may provide appropriate timing and an optimal frequency for MDA in Lao PDR. The current annual MDA is conducted in December, with consideration of the harvest season for rice (late November to December) and the season when most infection occurs (between April to June). Spatio-temporal changes in precipitation patterns due to climate change are expected to impact the number of schistosomiasis mekongi patients and may change the period and areas of high risk in the future. Precipitation has been increasing considerably since 2017 in the present study. Using Earth observation satellite data enables the collection of vast amounts of information including on climate change, and it is also considered extremely useful for determining many other infectious disease countermeasures.

The primary limitation of the present study is that only annual data were used because finer time scale data were not available. In addition, since schistosomiasis mekongi is a One Health disease, data on vector snails and mammals (such as dog and pig) [32,33] that can be infected with the parasite are urgently needed. A One Health approach must be utilized to effectively control and eliminate its transmission. The impact of precipitation on the prevalence of schistosomiasis is still in the early stages of research and will be further elucidated through monthly and more detailed data analysis, as well as additional information on vector snails and animals.

## 5 Conclusion

The results of the present study suggested that among climate change factors, attention to changes in precipitation should be considered in the policy to eliminate schistosomiasis mekongi in Lao PDR. It is also necessary to consider that precipitation is increasing, and the effect of this climate change works both positively and negatively on the prevalence of schistosomiasis mekongi in Lao PDR. Although an increase in yearly precipitation decreased the prevalence of schistosomiasis, conversely, the increase in precipitation in the dry season increased the prevalence of schistosomiasis. Earth observation satellite data provide enormous amounts of information that are extremely useful for determining measures taken against many infectious diseases including schistosomiasis.

## Funding

The present study was supported by a grant from the Earth Observation Research Center, 10.13039/501100004020Japan Aerospace Exploration Agency (JAXA) [17RSTK-006235, EO-RA1, EO-RA2, and EO-RA3], a grant from the JICA/AMED SATREPS project for “the development of innovative research technique in genetic epidemiology of malaria and other parasitic diseases in the Lao PDR for containing their expanding endemicity” (2013–2019) and a grant from the JICA/AMED SATREPS project for “Project for Malaria and Neglected Parasitic Diseases Control and Elimination using Advanced Research Technique, Communication Tools and Eco-Health Education” (2022–2028). The funding bodies have no role in the design of the study, data collection, analysis, and in writing the manuscript.

## Availability of data and materials

All data generated or analysed during the present study are included in this published article.

## Authors' contributions

ELAM-T, TK, SK, and MI drafted the study. ELAM-T and MI collected the data with the support of BH and PTB. Funding acquisition was by ELAM-T, SK, and MI. KO, YS, and YM helped to collect the satellite data and the analysis of these data. ELAM-T analysed the data and wrote the present paper under the supervision of TK, SK, and MI. All authors read and approved the final manuscript.

## Ethics approval

N/A.

## Consent for publication

N/A.

## Declaration of Competing Interest

The authors declare that they have no competing interests.

## Data Availability

The authors do not have permission to share data.
